# Personality portraits, resilience, and professional identity among nursing students: a cross-sectional study

**DOI:** 10.1186/s12912-024-02007-7

**Published:** 2024-06-21

**Authors:** Xiaona Wu, Yingzi Lu, Yihao Zeng, Hui Han, Xiaoming Sun, Jiapeng Zhang, Ning Wei, Zengjie Ye

**Affiliations:** 1 Department of Nursing, Guangzhou Women and Children Medical Center, Guangzhou Medical University Affiliated Women and Children Medical Center, Guangzhou, People’s Republic of China; 2 School of Nursing, Guangzhou University of Chinese Medicine, Guangzhou, People’s Republic of China; 3 Department of Health Management and Promotion, Guangdong Maoming Health Vocational College, Maoming, People’s Republic of China; 4 School of Marine finance and economics, Qingdao Engineering Vocational College, Qingdao, People’s Republic of China; 5 School of nursing, Medical College of Jiaying University, Meizhou, People’s Republic of China; 6 School of nursing, Guangzhou Xinhua University, Guangzhou, People’s Republic of China; 7 School of Nursing, Guangzhou Medical University, Guangzhou, Guangdong Province 511495 China

**Keywords:** Nursing students, Personality traits, Psychological resilience, Professional identity, Latent profile analysis, Mediation analysis

## Abstract

**Background:**

The lack of professional identity can impede the transition from nursing students to qualified nurses and exacerbate the shortage of health care professionals. Personality is important to resilience-building and professional identity development in nursing students. However, the associations among personality, resilience, and professional identity are less explored. The study aims to identify latent subtypes of personality, to evaluate the mediating role of resilience between personality and professional identity in nursing students, and to provide practical guidance for educators’ subsequent interventions with nursing students’ professional identity.

**Methods:**

1397 nursing students were recruited from Be Resilient to Nursing Career (BRNC) between October 2020 and April 2022 by cluster sampling from 4 universities in China. NEO Five-Factor Inventory, 10-item Connor-Davidson Resilience Scale, and Professional Identity Questionnaire for Undergraduate Students were administered. Analyses of latent profiles and mediations were performed.

**Results:**

Three latent personality types were identified: Over-sensitivity (35.4%), Ordinary (53.8%), and Flexibility (10.8%). Nursing role model was found to be a significant indicator of personality (Ordinary as ref, Over-sensitivity: OR = 0.73, 95% CI: 0.57–0.93, *P* = 0.010; Flexibility: OR = 1.85, 95% CI: 1.29–2.65, *P* = 0.001). The association between personality portraits and professional identity were significantly mediated by resilience (*P* < 0.05).

**Conclusions:**

There exists heterogeneity in nursing students’ personality. Resilience plays a significant role in mediating the relationship between personality and professional identity.

**Supplementary Information:**

The online version contains supplementary material available at 10.1186/s12912-024-02007-7.

## Background

Nursing students are at an important stage in their development of professional identity [1]. Nursing education, in Kantek’s opinion, is primarily concerned with preparing nursing students to acquire a sense of identity in terms of professional knowledge, values and competencies [2]. Professional identity refers to an individual’s acceptance and recognition of the profession he or she is studying, and is a process of continuous cognitive and positive state transfer during the learning process, in other words, a desire to learn and explore in a positive manner and a positive attitude [3, 4]. Nursing students with a stronger professional identity display more active learning behaviors and demonstrate greater development of professional competence. Meanwhile, they have a clear understanding of their career choices and are more inclined to continue in nursing after graduating [5]. However, there is, evidence that nursing students have a moderately low level of professional identity, resulting in reduced commitment to learning and career development [6, 7]. Therefore, it is imperative to improve their professional identity levels which will enhance the nursing workforce [8].

Personality comprises a set of relatively stable and unique psychological behavior patterns that determine a person’s attitude toward things as well as their behavior [9, 10]. Based on Holland’s vocational theory, individuals can become motivated and engaged in learning if their personality traits are in harmony with the requirements of their professions [11]. There is a strong correlation between personality type and professional identity, as evidenced by studies [12, 13]. However, most previous research has focused on a variable-centered approach to identify nursing students’ personality, which is poor at capturing individual specificity within and between cohorts [14]. Moreover, variable-centered approaches emphasize only one trait among personality traits, which makes it difficult to test and explain interactions among personality traits. The person-centered approach takes into account the whole person as they engage with their environment in order to provide a more coherent explanation of personality [14, 15]. Latent profile analysis (LPA) is used in many fields such as psychology and psychiatry, and its classification accuracy and validity are significantly higher than traditional classification [16]. As a result of LPA, different types of subgroups can be identified based on differences in exogenous variables, allowing for the capture of group inequalities that are not evident in variable-centered studies [17]. By means of LPA methods, it is possible to identify groups of individuals who exhibit a prototypical pattern of co-variation among personality traits, for example, subgroups scoring similarly on a number of particular personality traits [18]. This method is useful for obtaining conceptually useful profiles that assist in understanding the relationship between personality traits and other psychological concepts [19]. Therefore, it is worthwhile to explore the influence mechanism of personality portraits of nursing students on professional identity based on a person-centered approach.

**Fig. 1:**
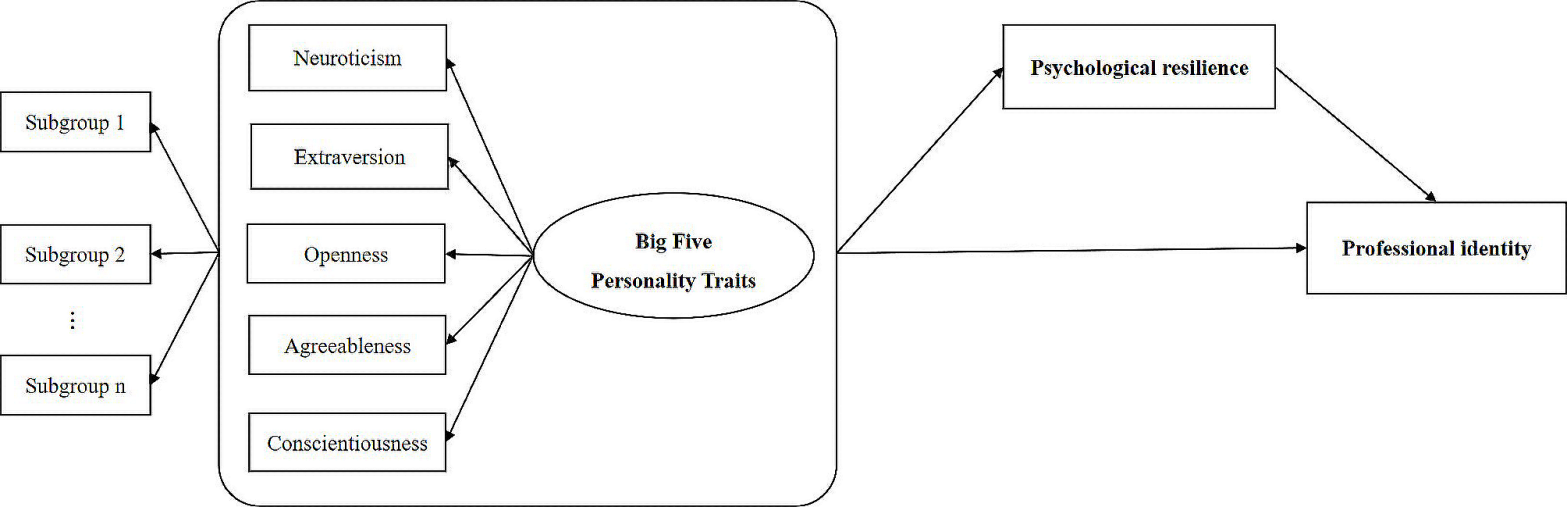
The hypothetical framework of big five personality traits, psychological resilience and professional identity among nursing students

Resilience is defined as a positive trait orientation or personality characteristics that enhances individual adaptation [20] according to Block’s [21] framework of personality development. From the perspective of positive psychology, personality traits play a significant role in the development of resilience [22, 23]. For example, personality trait was identified as a predictor of resilience and individuals with low neuroticism and high extraversion and conscientiousness were reported to have higher resilience [20, 24, 25]. Additionally, resilience plays a role in the process of professional identity formation (PIF) in medical students [25] and preliminary results confirmed that resilience was a significant predictor of professional identity in the educational environment [26, 27]. Thus, we have interests whether resilience plays a mediating role between personality portraits and professional identity. There have been previous studies on the influence of personality traits on professional identity that have focused on personality dimensional traits and their transmission channels, but little attention has been paid to the relationship between personality portraits, resilience, and professional identity. Thus, we have interests whether resilience plays a mediating role between personality traits and professional identity. We hypothesized that (Fig. 1):

### Hypothesis 1

Several potential personality portraits would be identified by LPA among nursing students.

### Hypothesis 2

Resilience might play a mediation role between LPA-based personality portraits and professional identity.

## Theoretical framework

This study was guided by self-identity theory, which was introduced to the field of psychology by Erikson [28]. The main mechanism for the constitution and differentiation of personality is identity, according to this theory. The trait of a normal personality is to have a stable sense of identity, such as a clear and well-defined sense of “who I am” and “what I want to be” [29, 30]. In the process of constructing a professional identity, which is subordinate to one’s self-identity, learners draw upon their own experiences and reflections to establish a holistic understanding of what distinguishes a particular discipline from others [31]. The process of forming internal attitudes toward the learned profession and role commitment is precisely what constitutes the construction of the learner’s self-identity [32]. Individuals may experience some psychological conflict during the construction process. However, some people are able to exercise self-control and adjust flexibly and are not easily dominated by negative emotions, which relies on psychological resilience [33]. Therefore, based on self-identity theory, the present study attempts to examine the relationship between personality portrait, resilience and professional identity from a person-centered perspective to lay a psychological foundation for enhancing nursing students’ professional identity.

## Methods

### Study design and sample

A multicenter cross-sectional design was adopted. By convenience sampling, 1522 nursing students were recruited from Be Resilient to Nursing Career (BRNC, a long-term project focused on personality, resilience, and career pathways among nursing students, Registration number: ChiCTR200003869) from four universities between October 2020 and April 2022 [26, 34–39]. The inclusion criteria were as follows: (1) majoring in nursing; (2) participation in this study is voluntary. Mental disorders diagnosed by clinical psychiatrists were excluded. Previous studies have indicated that Neuroticism has an effect of -0.21 on psychological resilience [36] and − 0.12 on professional identity [40]. And the effect of resilience on professional identity was found to be 0.42 [26] These path coefficients were substituted into the MedPower program (https://davidakenny.shinyapps.io/MedPower/) developed by Schoemann [41]. Desired power was set at 0.80, and Alpha for all power calculations set to 0.05. The minimum sample size required was 236 for the mediation model with a 20% dropout rate. A total of 1423 participants filled out the booklet and a response rate of 93.50% was achieved. Data were excluded from participants with missing records (27/1423, 1.90%). A checklist for reporting observational studies (STROBE) was used for the reporting of this study (see Supplementary file S1).

An offline survey was used to collect questionnaires. The lead researcher contacted the counselors at four universities, who informed the students of the survey’s content, time, and location. All students were invited to participate in the study under the counselor’s jurisdiction. At the survey meeting, the lead researcher presented a presentation to explain the study’s purpose, content, and significance, as well as to answer participants’ questions. After informed consent was obtained from the participants, the trained researchers administered the questionnaire. The questionnaire was self-administrated and was checked as soon as being returned. Trained researchers would explain and ask participants to finish the blank, if any. Approximately 15 to 25 min were required to complete the questionnaire. A small gift was given to participants following the data collection (one notebook with a size of 13.8 × 20.5 cm and two black marker pens).

### Measures

#### Demographics characteristics

In accordance with previous research, demographic and professional-related characteristics of participants were collected: age, sex, hometown, and nursing role model [26, 34, 35, 42, 43].

#### Measurement of personality traits

The NEO Five-Factor Inventory (NEO-FFI) is used to assess personality traits that contains 60 items on five dimensions including neuroticism, extraversion, openness, agreeableness and conscientiousness [44]. Each of the five personality domains consist of 12 items, giving the total score of 60 points. Cronbach’s alpha for domains ranged from 0.618 to 0.833 in the present study.

#### Measurement of resilience

A Chinese version of 10-item Connor-Davidson Resilience Scale (CD-RISC-10) is a generic resilience instrument [45]. The questionnaire contains 10 items and is scored from 0 to 40, with higher scores indicating greater resilience. The Cronbach’s alpha of 0.879 was identified for CD-RISC-10 in this study.

#### Measurement of professional identity

A Chinese Version Professional Identity Questionnaire for Undergraduate Students (PIQUS) was developed by Qin [46], containing 23 items spanning four dimensions. There is a wide range of achievement scores for PIQUS (from 23 to 115 on a Likert scale of 5), with higher scores indicating a higher level of professional identity [34]. The overall Cronbach’s alpha coefficient in the present study was 0.936.

### Data analyses

First, descriptive statistics were described as frequencies, percentages, means and standard deviations. The differences between subgroups were analyzed using independent sample t-tests. Second, the associations among personality, resilience, and professional identity were assessed using Spearman correlation analysis. Third, in order to identify latent subgroups with distinct personalities, LPA was conducted. The process started with a one-class model and evolved until the fit indices could not be significantly improved. Akaike Information Criterion (AIC), Bayesian Information Criterion (BIC), sample size-adjusted BIC (aBIC), Entropy value, and Lo–Mendell–Rubin likelihood ratio test (LMRT) [47] were used to determine the optimal model. Using AIC, BIC, and aBIC, an evaluation of model fit is made by taking the difference between the expected value and the actual value (a smaller value indicates better model fit) [48]. Lo-Mendell-Rubin (LMR) was used to evaluate the fitting differences among potential profile models. If the P-value reached the significance level, the model with k categories was significantly superior to the model with k-1 categories. A higher Entropy value (on a scale of 0 to 1), closer to 1, indicates a higher classification accuracy [47, 49]. Other considerations include adequate sample size and clinical significance of the underlying category. We took theory, parsimony, interpretability, false discovery rate (FDR) [50] into consideration to select a final model. Each subgroup should contain at least 10% of the sample size in order to avoid false-positive classification [51, 52]. Moreover, the potential indicators of LPA-based personality were also evaluated through univariate (*P* < 0.2) [53] and multivariate logistic regressions. Fourth, the professional identity was compared with different LPA-based personality portraits using Bayesian ANOVA and post-hoc analysis. The Bayes factor value greater than 10 indicates strong relative evidence for a hypothesis [54]. Lastly, resilience was estimated as a mediator between LPA-based personality portraits and professional identity. Data processing tools were SPSS 22.0, Mplus 8.3, and JASP 0.16.0. Significance was set at 0.05.

### Ethical considerations

The study was approved by the Ethics Committee of the First Affiliated Hospital of Guangzhou University of Traditional Chinese Medicine (No: ZYYEC-ERK【2020】132). Informed consent was obtained from all participants after verbal explanation of the procedure and its purpose, which adhered to the Declaration of Helsinki. Data privacy and anonymity were reassured to the participants.

## Results

### Demographic characteristics

The total sample consisted of 1397 nursing students who were mainly female 1048 (65.0%). 984 subjects (70.4%) received a bachelor education, while the others received a junior college education (29.6%). 17.5% of the students reported being the only child in their family. 382 (27.3%) of students had relatives who were medical staff and 830 students (59.4%) reported having a role model in nursing. There were 447 (32.0%) students who considered leaving their profession.

### Correlation analysis of personality, resilience, and professional identity

**Fig. 2:**
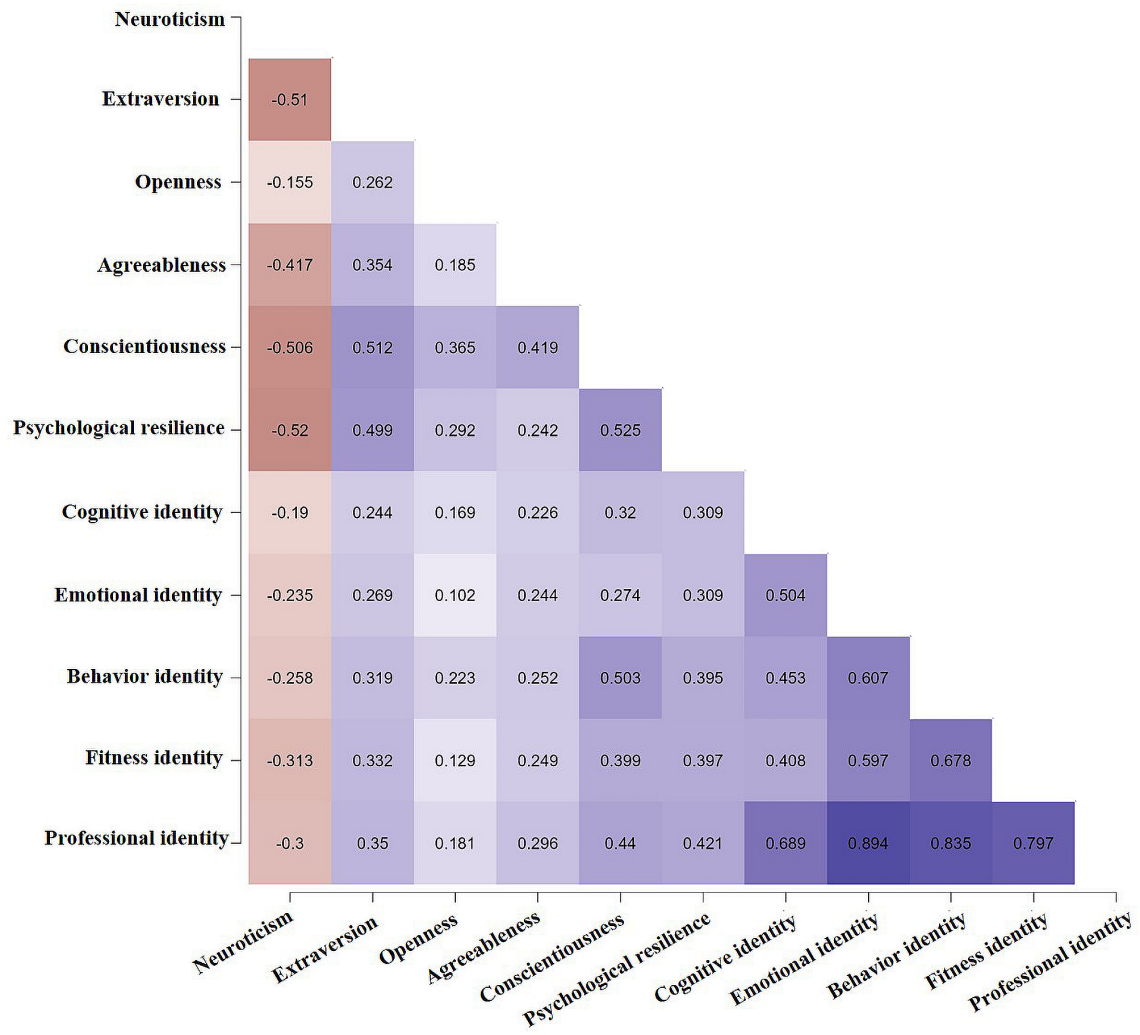
Pearson correlation Heatmap among big five personality traits, psychological resilience and professional identity

The mean and standard deviations of variables were resilience (25.18 ± 5.94), professional identity (84.91 ± 13.92), neuroticism (32.95 ± 7.86), extraversion (39.51 ± 5.73), openness (41.83 ± 5.46), agreeableness (43.10 ± 5.46), and conscientiousness (42.71 ± 6.20). Fig. 2 illustrates Pearson’s correlation heatmap (all *P* < 0.001). Neuroticism was negatively associated with resilience (*r* = -0.52). Psychological resilience was positively associated with conscientiousness (*r* = 0.53) and professional identity (*r* = 0.42).

### Latent profiles analysis of personality traits

**Fig. 3:**
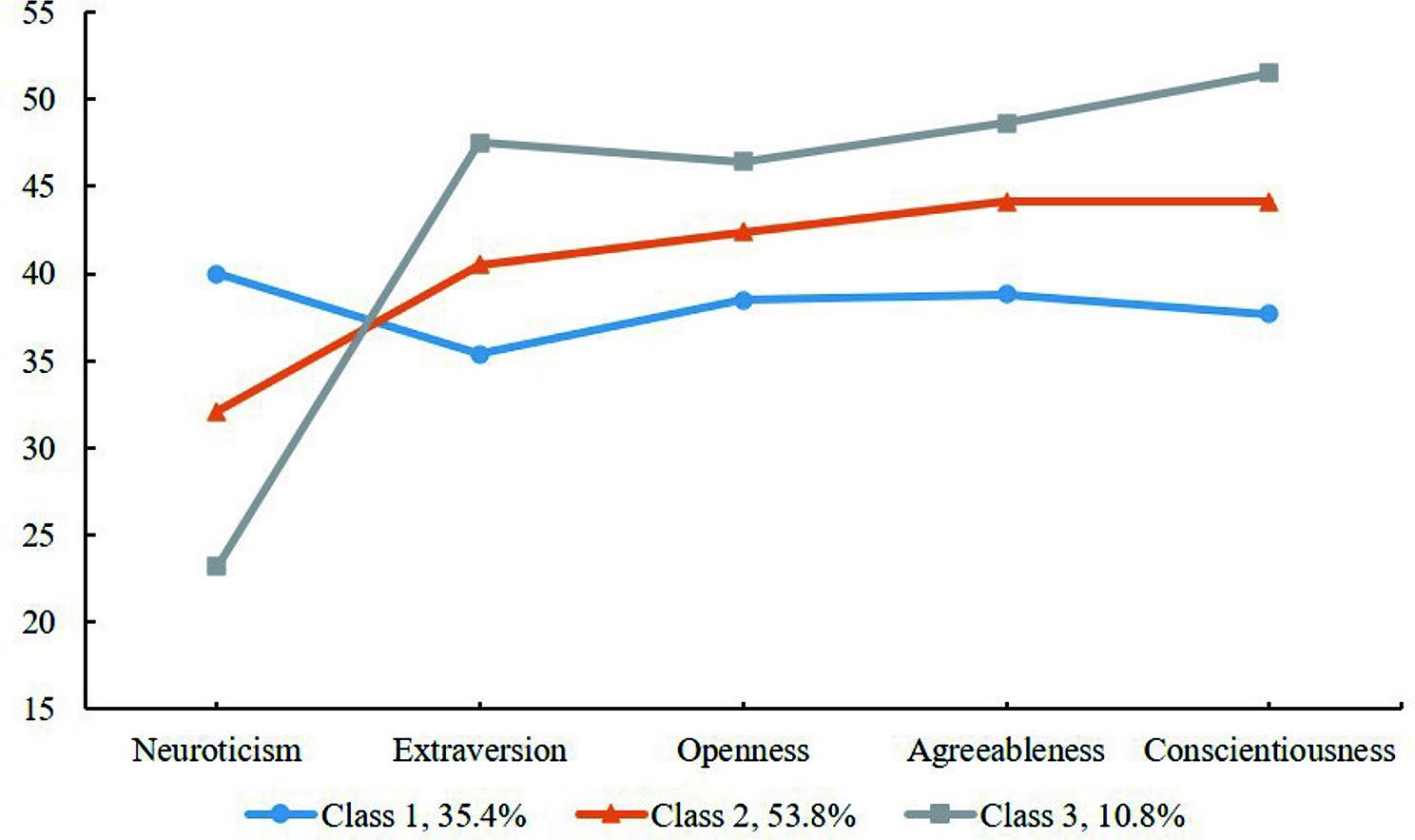
Parameters for the final three-class patterns. C1 = Over-sensitivity, C2 = Ordinary; C3 = Flexibility

The best models were found to be of three types based on theory, parsimony, and fitting indicators. Table 1; Fig. 3 details additional information. Three personality portraits were identified: Over-sensitivity (35.4%, Class 1, be supersensitive to situational cues), Ordinary (53.8%, Class 2, normative and unremarkable characteristics of personality), and Flexibility (10.8%, Class 3, adaptive response system with resilience). Hypothesis 1 was supported. Using logistic regression (see Table 2), only nursing role model was indicative of personality portraits (OR = 0.73, 95% CI: 0.57–0.93, *P* = 0.010; OR = 1.85, 95% CI: 1.29–2.65, *P* = 0.001) after controlling for covariates.

**Table 1:** Fitting Index and Group Size of Latent Profile Analysis Models

Indicators			LPA Model		
	1-Class	2-Class	3-Class	4-Class	5-Class
Fit statistics					
*LL*	-22456.90	-21890.84	**-21741.74**	-21675.14	-21636.50
*AIC*	44933.79	43813.68	**43527.48**	43406.28	43341.00
*BIC*	44986.21	43897.55	**43642.80**	43553.06	43519.232
*aBIC*	44954.45	43846.72	**43572.92**	43464.11	43411.227
*Entropy*	1.000	0.708	**0.712**	0.734	0.741
*LMR (P)*	—	< 0.001	**0.001**	< 0.001	0.117
Group size (%)					
C1	1397 (100.0)	856 (61.3)	**495 (35.4)**	113 (8.1)	111 (7.9)
C2	—	541 (38.73)	**751 (53.8)**	498 (35.6)	666 (47.7)
C3	—	—	**151 (10.8)**	698 (45.0)	62 (4.4)
C4	—	—	**—**	88 (6.3)	92 (6.6)
C5	—	—	**—**	—	466 (33.4)

*Note* Bold figures highlight the selected class solution

**Table 2:** Univariate and multivariate logistic regression results for predicting external features on the 3-class pattern

Variables	LPA-based personality portrait types								
	Univariate analysis					Multivariate analysis			
	Over-sensitivity vs. Ordinary		Flexibility vs. Ordinary			Over-sensitivity vs. Ordinary		Flexibility vs. Ordinary	
	OR (95%CI)	***P***	OR (95%CI)	***P***		OR (95%CI)	***P***	OR (95%CI)	***P***
Gender (female as ref)	1.04 (0.80–1.35)	0.764	1.11 (0.75–1.65)	0.606					
Educational level (junior college as ref)	0.88 (0.69–1.13)	0.310	1.01 (0.68–1.48)	0.970					
Only children (no as ref)	1.09 (0.81–1.46)	0.587	0.68 (0.40–1.13)	0.136		1.07 (0.80–1.44)	0.641	0.69 (0.41–1.17)	0.167
Place of residence (countryside as ref)	0.93 (0.73–1.17)	0.517	1.04 (0.73–1.50)	0.817					
Any medical staffs as relatives (no as ref)	0.92 (0.71–1.19)	0.507	1.19 (0.81–1.74)	0.375					
Nursing role model (no as ref)	0.72 (0.57–0.92)	0.007	1.91 (1.34–2.73)	< 0.001		**0.73 (0.57–0.93)**	**0.010**	**1.85 (1.29–2.65)**	**0.001**
Willingness to leave the profession (no as ref)	1.11 (0.87–1.41)	0.394	0.75 (0.50–1.11)	0.146		1.05 (0.82–1.34)	0.717	0.85 (0.57–1.28)	0.434

*Abbreviations* OR, Odds Ratio; CI, Confidence Interval. Bold figures highlight statistically significant in the multivariate logistic regression

### LPA-based personality differences on professional identity scores

**Fig. 4:**
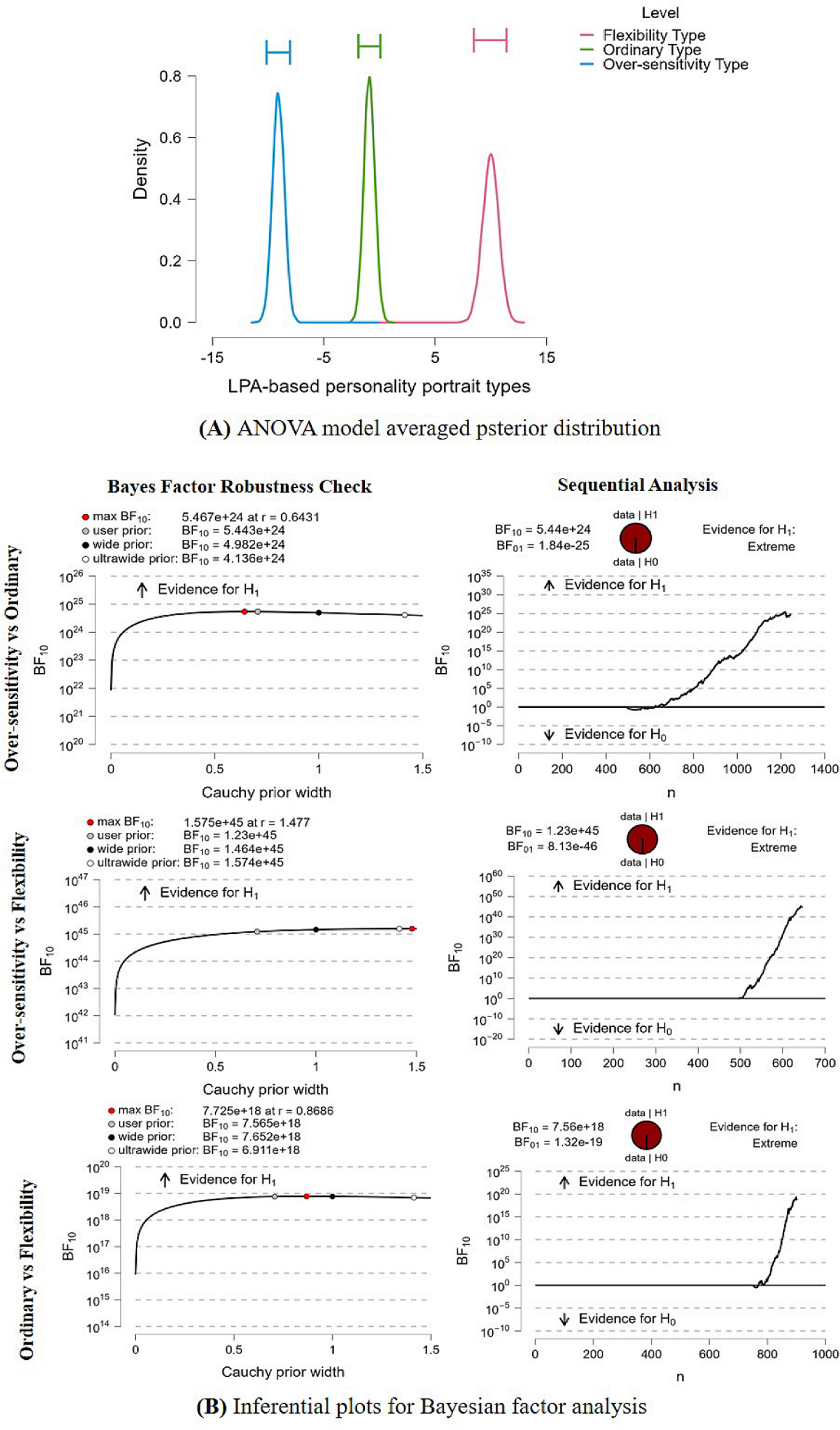
(A) ANOVA model averaged psterior distribution; (B) Inferential plots for Bayesian factor analysis

As shown in Table 3, between-group differences (F = 148.929, *P* < 0.001) in personality portraits were significant in the professional identity scores between Over-sensitivity and Ordinary (BF_10_ = 5.44e^24^, d = -0.65), between Over-sensitivity and Flexibility (BF_10_ = 1.23e^45^, d = -1.49), and between Ordinary and Flexibility (BF_10_ = 7.57e^18^, d = -0.88). These findings were confirmed by Bayesian Factor Robustness analysis, which was presented in Fig. 4.

**Table 3:** ANOVA comparisons of professional identity scores across LPA-based personality traits types and post-hoc comparisons by Bayesian factor analysis

LPA-based differences in professional identity scores					
**Subgroups**	**N**	**M ± SD**	***F***	***P***	${\varvec{\eta }}^{2}$
Over-sensitivity	495	78.42 ± 12.96	148.929	< 0.001	0.176
Ordinary	751	86.63 ± 12.38			
Flexibility	151	97.65 ± 12.88			
**Bayesian post-hoc comparisons**					
**Groups**	***MD (95%CI)***		***Cohen’s d***	***BF***_***10***_	**error%**
Over-sensitivity vs. Ordinary	-8.21 (-9.65, -6.78)		-0.65	5.44e^24^	8.06e^− 28^
Over-sensitivity vs. Flexibility	-19.23 (-21.60, -16.87)		-1.49	1.23e^45^	1.29^e − 50^
Ordinary vs. Flexibility	-11.02 (-13.20, -8.84)		-0.88	7.57e^18^	7.68^e − 26^

*Note* LPA-based personality portrait differences in professional identity scores in Bayesian Factor. ${\eta }^{2}$= eta squared represents variance of a dependent variable by three LPA-based subgroups

*Abbreviations* M, Mean; SD, Standard Deviation; MD, Mean Differences; BF, Bayes Factor

### Mediation analysis of resilience between LPA-based personality and professional identity

**Fig. 5:**
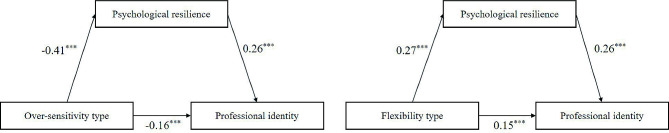
A hypothesized mediator model with three LPA-based personality portrait types as independent variable (**X**), psychological resilience as a mediator (**M**), and professional identity as dependent variable (**Y**). Ordinary type is used as the reference. The control variables are not presented in the figure for brevity

As shown in Tables 4 and 5, Ordinary group was taken as reference, the indirect effect (-0.11), direct effect (-0.16) and total effect (-0.26) with 95% Bootstrap confidence intervals of (0.09, 0.21), (0.19, 0.42), (0.29,0.66), respectively, indicating that resilience significantly mediated the relationship between Over-sensitivity group and professional identity, achieving a 40.74% mediating effect. Similarly, the indirect effect (0.05, 0.09), direct effect (0.10, 0.19) and total effect (0.17, 0.26) indicated that resilience significantly mediated the relationship between Flexibility group and professional identity, with a mediating effect of 33.33%. Statistically, the model was responsible for 42.80% of the variance in professional identity (*P* = 0.001). Thus, Hypothesis 2 was verified. There was an advance control of all confounding factors. Further details are provided in Fig. 5.

**Table 4:** The mediating effect of sense of coherence on professional identity

Variables	β	SE	t	*P*	LLCI	ULCI	*R* ^2^
	**Mediating variable model (Outcome variable: Psychological resilience)**						0.302
Over-sensitivity type	-0.41	0.02	-19.01	< 0.001	-0.45	-0.37	
Flexibility type	0.27	0.02	11.27	< 0.001	0.23	0.32	
	**Dependent variable model (Outcome variable: Professional identity)**						0.418
Over-sensitivity type	-0.16	0.02	-6.64	< 0.001	-0.21	-0.11	
Flexibility type	0.15	0.02	6.36	< 0.001	0.10	0.19	
psychological resilience	0.26	0.03	9.00	< 0.001	0.20	0.31	

*Note* Any medical staffs as relatives, nursing role model, and willingness to leave the profession are controlled statistically

Ordinary type is used as the reference

*Abbreviations* SE, Standard Error; LLCI, Lower Confidence Interval; ULCI, Upper Confidence Interval

**Table 5:** Direct and indirect effect of personality portraits on professional identity

Model pathways	Effect	SE	t	LLCI	ULCI
Indirect effect					
Over-sensitivity type → resilience → professional identity	-0.11	0.01	-8.14	-0.13	-0.08
Flexibility type → resilience → professional identity	0.07	0.01	6.94	0.05	0.09
Direct effect					
Over-sensitivity type → professional identity	-0.16	0.02	-6.64	-0.21	-0.11
Flexibility type → professional identity	0.15	0.02	6.36	0.10	0.19
Total effect					
Over-sensitivity type → resilience → professional identity	-0.26	0.02	-11.49	-0.31	-0.22
Flexibility type → resilience → professional identity	0.21	0.02	9.53	0.17	0.26

*Abbreviations* SE, Standard Error; LLCI, Lower Confidence Interval; ULCI, Upper Confidence Interval

## Discussion

First, three personality portraits were identified among nursing students, including Over-sensitivity, Ordinary, and Flexibility. These findings were similar to the profiles found in Udayar at al.’s [19] and Wentao at al.’s [55]. More attentions should be paid to nursing students with Over-sensitivity portrait (35.4%) as this subgroup exhibits more socially undesirable personality characteristics as compared to the other two profiles [19]. In this case, Over-sensitivity portrait may be interpreted as reinforcing the perception of the negative aspects of nursing profession [14]. Moreover, it was observed that nursing students who had nursing role models were more likely to be assigned to Flexibility group than those who did not. Role modeling is an integral component of medical students’ values, attitudes, and professional character [56, 57]. Nursing students imitate and learn the professionalism and professional values of role models, which is a process of internalization and self-regulation [58, 59]. Hence, role modeling is considered as the core of shaping their personality [56]. Role modeling is considered as an effective teaching method that a positive learning environment and role modeling educational programs [56] can assist nursing students in honing their clinical skills and developing their personality.

In this study, Flexibility group had the highest levels of professional identity, while the Over-sensitivity group had the lowest. Students with over-sensitivity are more likely than others to detect a variety of emotional triggers due to their heightened sense of situational cues (i.e., professional pressure and social evaluation, etc.) [56]. Their perceptions of the profession are easily disturbed by the external environment, such as social prejudice in China [60] and nurse-patient relationships [61], which reinforce their negative emotions. Gradually, they begin to deny and doubt about nursing profession [62]. On the contrary, nursing students with Flexibility enable them to approach the occupational environment with rationality and resilience [63], resulting in a clear career perception as well as a high level of loyalty to the nursing profession [64].

Third, it was reported that resilience played an important role in mediating the association between personality portraits and professional identity among nursing students. Our hypothesis framework was accepted in the present study (Fig. 1). In this study, nursing students in Over-sensitivity displayed the highest level of neuroticism, which was associated with high levels of stress perception [36] and emotional coping [65]. As a result, they coped with stressful situations and negative evaluations in a fragile manner compare to Ordinary profile. Resilience can prevent stressors that lead to the erosion of core values [66], thus reducing risk of depersonalization, academic burnout, and loss of identity [25]. However, nursing students with Over-sensitivity had low levels of positive traits (e.g., openness, conscientiousness, agreeableness, etc.) which are related to resilience-building [20]. Therefore, they are unable to develop attitudinal patterns for coping or motivation [40], resulting in a lack of confidence in their professional identity. Conversely, compared to the Ordinary profile, nursing students in the Flexibility were more cognitively and emotionally identified with the nursing profession, accompanied by positive internal evaluations and external behaviors [55]. They are capable of adjusting to changes in situational cues and are more rational about their work environment. Moreover, the nursing profession is more positively viewed by them, leading to greater loyalty to the profession [63].

In conclusion, we found that the personality traits of nursing students were heterogeneous based on LPA, which means that different groups of nursing students present different personality traits. Additionally, resilience mediates the relationship between personality portraits and professional identity. Personality has a profound influence on nursing students’ thoughts, feelings and behavior, and plays an important role in building psychological resilience [40]. As an integral component of PIF, resilience is also known as “emotional competence.” [25]. In detail, resilience may diminish the effect of negative personality traits and enhance emotional experience of positive traits [67]. According to Broaden-and-Build theory, positive emotions enable individuals to create lasting personal resources, including intellectual and psychological resources, to deal with future challenges [68]. Thus, personality traits may influence resilience by changing an individual’s approach to evaluating stressors, thereby enabling them to be more flexible with their cognitive processes and professional identity [68]. This could be a potential mechanism for the mediating role of resilience in the relationship between personality traits and professional identity. There should be more attention paid to nursing students with oversensitive personalities. Some preventive interventions could be developed for this vulnerable group. Resilience-building exercises [1] should be considered to build into the curriculum to promote students’ resilience. It has been established that flexibility/strength exercise treatment (FLEX) [34, 69] may enhance resilience. Role modeling is a complex and multifaceted process that requires accurate perceptions, as well as clinical nursing knowledge and skills for educators [56, 70].

### Limitations

Some limitations should be considered. First, A consideration of the differences in cultural background should be taken into account when extrapolating the findings. Second, considering that the current study is cross-sectional, it is not feasible to establish a causal relationship. Therefore, it would be beneficial to conduct a longitudinal study to confirm the findings. In the future, more insights will be provided by our ongoing cohort (BRNC).

## Conclusions

Students in nursing are heterogeneous in terms of their personalities. The association between personality portraits and professional identity is mediated by resilience. Nursing educators and administrators should identify nursing students’ personality portraits early on and develop interventions to enhance their resilience and professional identity.

### Electronic supplementary material

Below is the link to the electronic supplementary material.

Supplementary Material 1

## Data Availability

The data that support the findings of this study are available from the corresponding author upon reasonable request.
